# Mechanistic Drivers of Flexibility in Summit Metabolic Rates of Small Birds

**DOI:** 10.1371/journal.pone.0101577

**Published:** 2014-07-03

**Authors:** David Swanson, Yufeng Zhang, Marisa King

**Affiliations:** Department of Biology, University of South Dakota, Vermillion, South Dakota, United States of America; Pennsylvania State University, United States of America

## Abstract

Flexible metabolic phenotypes allow animals to adjust physiology to better fit ecological or environmental demands, thereby influencing fitness. Summit metabolic rate (M_sum_ = maximal thermogenic capacity) is one such flexible trait. Skeletal muscle and heart masses and myocyte metabolic intensity are potential drivers of M_sum_ flexibility in birds. We examined correlations of skeletal muscle and heart masses and pectoralis muscle citrate synthase (CS) activity (an indicator of cellular metabolic intensity) with M_sum_ in house sparrows (*Passer domesticus*) and dark-eyed juncos (*Junco hyemalis*) to determine whether these traits are associated with M_sum_ variation. Pectoralis mass was positively correlated with M_sum_ for both species, but no significant correlation remained for either species after accounting for body mass (M_b_) variation. Combined flight and leg muscle masses were also not significantly correlated with M_sum_ for either species. In contrast, heart mass was significantly positively correlated with M_sum_ for juncos and nearly so (*P* = 0.054) for sparrows. Mass-specific and total pectoralis CS activities were significantly positively correlated with M_sum_ for sparrows, but not for juncos. Thus, myocyte metabolic intensity influences M_sum_ variation in house sparrows, although the stronger correlation of total (*r* = 0.495) than mass-specific (*r* = 0.378) CS activity with M_sum_ suggests that both pectoralis mass and metabolic intensity impact M_sum_. In contrast, neither skeletal muscle masses nor pectoralis metabolic intensity varied with M_sum_ in juncos. However, heart mass was associated with M_sum_ variation in both species. These data suggest that drivers of metabolic flexibility are not uniform among bird species.

## Introduction

Phenotypic flexibility refers to reversible adjustments of the phenotype (i.e., physiology, morphology or behavior) to prevailing environmental or ecological conditions [1]. Such flexibility allows organisms to better match phenotypes to energetic or ecological demands and may have fitness consequences [2]. Both minimum and maximum metabolic outputs in birds show substantial phenotypic flexibility in response to varying environmental or ecological demands [3]–[5]. Summit metabolic rates (M_sum_ = maximum cold-induced metabolic rates) are positively correlated with cold tolerance in birds [6], [7] and positively correlated with overwinter survival in endotherms [8]–[10], suggesting positive fitness consequences for high M_sum_. Moreover, M_sum_ is broadly correlated with geographic ranges in birds, with high M_sum_ in taxa inhabiting regions with cold winter climates [11]–[13], suggesting that high M_sum_ is an important determinant of the ability to overwinter in cold climates.

M_sum_ is typically higher in winter than in summer for small birds wintering in cold climates, with summer to winter increases generally ranging from 10–50% [5]. Such winter increments of M_sum_ are correlated with improved winter cold tolerance [14]–[18]. Providing additional evidence for temperature being the selective factor driving flexibility in M_sum_, the opposite pattern, higher M_sum_ in summer than in winter, is often observed for small birds wintering in warm winter climates where winter thermoregulatory demands are relaxed [19], [20]. Summer increments of M_sum_ in these species probably occur a by-product of higher activity levels and daily energy expenditures in summer than in winter for these species [19], [20]. The migratory phenotype is also associated with increments of M_sum_ of 10–25% in several bird species [21]–[24]. However, these increments may be a by-product of adjustments for endurance flight rather than for thermogenesis [22], [24], although thermogenic benefits may accrue nonetheless. Indeed, Petit and Vézina [25] recently demonstrated experimentally that increasing flight costs of black-capped chickadees (*Poecile atricapillus*) improved thermogenic performance and M_sum_. Collectively, these studies show that M_sum_ is a flexible physiological trait that varies in a manner consistent with expected fitness consequences.

Summit metabolic rates are primarily a function of skeletal muscle shivering in birds and because the flight muscles are the largest muscle group in birds, these (especially the pectoralis muscle) serve as the primary thermogenic organ [26]. Thus, flexibility in M_sum_ should be associated with flexibility in either the mass or the cellular metabolic intensity of the pectoralis muscle. In addition, because prolonged high-intensity shivering requires enhanced delivery of oxygen and substrates to the shivering muscles, cardiovascular adjustments, including changes in heart mass, may accompany pectoralis muscle adjustments [5]. Several studies have examined seasonal variation in skeletal muscle and heart masses, and these studies typically find larger pectoralis and heart muscle masses in winter relative to summer [17], [27]–[31], although this generalization is not without exceptions [32], [33]. In addition, correlations of residual variation in pectoralis muscle size with residual variation in M_sum_ for individual birds are generally positive [23]–[25], [34], [35]. Similarly, pectoralis muscle mass is also positively associated with maximum exercise-induced metabolic rates in birds [36], [37]. Significant positive correlations of heart or cardiopulmonary masses with maximum exercise metabolic rates or M_sum_ also occur for several species [35]–[37]. Thus, pectoralis muscle and heart masses are prominent effectors of maximum metabolic output in birds, suggesting that variation in these traits may drive M_sum_ flexibility.

Flexibility in M_sum_ could also be driven, independent of and/or in addition to skeletal muscle and heart mass changes, by variation in cellular metabolic intensity, particularly within the pectoralis muscle. Activities of key aerobic regulatory enzymes, such as citrate synthase (CS) and cytochrome c oxidase (COX), are commonly used as metrics of cellular metabolic intensity in birds [5]. Activities of these enzymes increase with winter-acclimatization or migration in a number of species [5], [26], but remain seasonally stable for a number of other species [30], [38], [39]. Pectoralis CS activity was positively correlated with maximum exercise metabolic rates among individual male red junglefowl (*Gallus gallus*), but this relationship did not occur for females [37]. In addition, pectoralis CS activity was not significantly correlated with M_sum_ for individual American goldfinches (*Spinus tristis*) [34] and maximum exercise metabolic rate in house sparrows was better explained by pectoralis mass than by total pectoralis CS activity [36], [40]. Thus, variation in cellular metabolic intensity may contribute to M_sum_ flexibility in some bird species, but not in others. As such, its role as a driver of M_sum_ flexibility appears less consistent than that of variation in pectoralis muscle and heart masses.

To investigate whether muscle mass, cellular metabolic intensity, or both, are prominent and consistent drivers of M_sum_ variation in small birds, we examined correlations of M_sum_ with pectoralis muscle mass, combined flight and leg muscle masses, heart mass, and pectoralis CS activity (as a measure of cellular metabolic intensity) in dark-eyed juncos (*Junco hyemalis*) and house sparrows (*Passer domesticus*). Both of these species winter in cold climates and show winter increments of M_sum_ and cold tolerance relative to summer that are accompanied by winter increases in pectoralis muscle mass [7], [15], [27], [29], [31]. House sparrows also show winter increments of heart mass [29], but seasonally stable mass-specific pectoralis muscle cellular metabolic intensity [38]. We hypothesized that pectoralis muscle and heart masses would be positively correlated with M_sum_ in both species, but that other muscle masses would contribute little to M_sum_ variation due to their smaller sizes and more peripheral location, away from central vital organs. We also hypothesized that pectoralis cellular metabolic intensity would be positively correlated with M_sum_, although perhaps less tightly than pectoralis muscle and heart masses.

## Materials and Methods

### Bird Collection and Housing

We conducted this study under approval from the University of South Dakota Institutional Animal Care and Use Committee, protocol 79-01-11-14B, and captured birds under active federal (MB758442) and state (11-7, 12-2 and 13-4) scientific collecting permits. All procedures in the study conformed to the Ornithological Council’s *Guidelines to the Use of Wild Birds in Research* [41]. We captured all birds by mist net or live trap near Vermillion, Clay County, South Dakota (approximate latitude 43°N). We held both study species captive for at least three weeks prior to measurements, but captured the two species at different seasons and did not hold birds under identical captive conditions. However, we have no reason to suspect that differential captivity conditions should alter the fundamental relationships among metabolic rates, organ masses and cellular metabolic intensity in the two study species. We captured dark-eyed juncos in December of 2011 and 2012 and housed birds under four different 6-week temperature/photoperiod treatments (24°C, 8L:16D; 24°C, 16L:8D; 3°C, 8L:16D; 3°C, 16L:8D) [42] until late February or early March, when we conducted experiments. We captured house sparrows in mid-late May of 2011, 2012 and 2013, and held birds captive for 3–4 weeks at room temperature (23°C) and a 12L:12D photoperiod prior to measurements. We provided captive birds with mixed wild bird seed, a protein supplement (consisting of a homogenized mixture dry dog food with a minimum protein content of 21% and hard-boiled eggs), and vitamin-enriched water (Wild Harvest Multi-Drops vitamin supplement for all birds, United Pet Group, Inc., Cincinnati, OH) *ad libitum*. We also provided sparrows with 6 mealworms (*Tenebrio* larvae) per day.

### M_sum_ Measurement

We measured M_sum_ on the day before euthanasia and tissue dissections to allow birds to recover from the cold exposure treatment. We measured M_sum_ by open-circuit respirometry using a 1.9 L metabolic chamber designed from a half-gallon paint can with the inside painted flat black to facilitate loss of heat produced by the bird to the outside of the chamber. For M_sum_ measurements, we used a sliding cold exposure protocol [43] in a helox (79% helium/21% oxygen) atmosphere to facilitate heat loss without impairing oxygen extraction [44]–[46]. We controlled chamber temperature by immersing the metabolic chamber into a bath of water/ethylene glycol (Forma Scientific Model 2095, Marietta, OH, USA) capable of regulating temperature within ±0.2°C. After introducing the bird into the metabolic chamber, but before immersing the chamber into the water/ethylene glycol bath, we first flushed the chamber with helox for 5 min to replace air with helox.

Following immersion of the metabolic chamber into the bath, we recorded excurrent oxygen concentration every 5 sec with an Ametek S-3AII Oxygen Analyzer (Pittsburgh, PA, USA) using Datacan 5.0 data collection software (Sable Systems, Henderson, NV, USA). We calibrated the oxygen analyzer with dry, CO_2_-free room air each day prior to M_sum_ measurements. We maintained flow rates of dry, CO_2_-free helox at 1020±10 ml O_2_ min^-1^ throughout metabolic measurements with a Cole-Parmer Model Precision Rotameter (Model FM082–03ST, Chicago, IL, USA) calibrated with a soap bubble meter to ±1% accuracy. The temperatures at the beginning of the helox cold exposure were –2 to –5°C for juncos and –5 to –9°C for sparrows. We maintained the chamber temperature at the initial test temperature for 20 min and decreased temperature approximately 3°C every 20 min thereafter until the individual bird showed a steady decline in oxygen consumption over several minutes indicative of hypothermia. After removal of the bird from the metabolic chamber, we measured body temperature with a thermocouple thermometer (Cole-Parmer Model 8500-40, Chicago, IL, USA) and lubricated 20-gauge copper-constantan thermocouple inserted into the cloaca to a depth of approximately 1 cm, where further insertion did not affect the temperature reading. We considered birds with body temperatures ≤37°C at the end of the metabolic trial as hypothermic, and birds were invariably hypothermic at the end of metabolic trials, thereby verifying that thermogenic capacity had been reached. M_sum_ trials generally lasted from 30–75 min. We weighed birds to the nearest 0.1 g both before and after M_sum_ measurements and assumed constant mass loss over the measurement period. We then calculated M_b_ for the period of the cold exposure where M_sum_ occurred and used this value for M_b_ in later analyses. All M_sum_ measurements were conducted during the day, between 1000–1700 CST.

We calculated oxygen consumption rates from excurrent oxygen content measurements with ExpeData 2.0 software (Sable Systems, Henderson, NV, USA), using the instantaneous correction [47]. We calculated running 5-min mean oxygen consumption values over the course of the metabolic measurements, and considered the highest 5-min mean as summit metabolic rate [11], [48]. We corrected all values of oxygen consumption to STPD.

### Tissue Dissections and Citrate Synthase Assays

On the day following M_sum_ measurement, we euthanized birds by cervical dislocation, rapidly plucked feathers from the underparts, and excised skeletal muscles and hearts with the bird resting on ice. After excision, we weighed the wet mass of right pectoralis, right supracoracoideus, right mixed leg muscles (thigh and gastrocnemius), and heart to the nearest 0.0001 g and then dropped tissues in liquid nitrogen for flash-freezing. Dissections were completed within 20–30 minutes of euthanasia. We stored tissues at −80°C until later enzyme assays. For later calculations requiring overall muscle masses for paired muscles, we used the mass of the right muscles multiplied by two.

We measured citrate synthase (CS, E.C. 4.1.3.7) activity in pectoralis muscles as an indicator of maximal cellular metabolic intensity [38], [49], [50]. For assays of pectoralis CS activity, we removed small samples of frozen tissues, minced them and diluted them in 10–40 volumes/mass of homogenization buffer (100 mM phosphate buffer with 2 mM EDTA at pH 7.3). We homogenized tissues on ice with short bursts (several seconds) at high speed using a Tekmar Tissuemizer (Model ST-1810, Cincinnati, OH, USA). Following homogenization, we sonicated tissues on ice for three 10 sec bursts, with 30 sec between bursts, with a Cole-Parmer (Chicago, IL, USA) 4710 Series Ultrasonic homogenizer. We used crude homogenates following sonication for enzyme assays.

The assay buffer contained 100 mM triethanolamine-HCl, 2.5 mM EDTA, 0.1 mM 5.5′-dithiobis-(2-nitrobenzoic acid), 0.2 mM acetyl-CoA, and 0.5 mM oxaloacetate (omitted for control) at pH 7.5 in a final volume of 1.0 mL [38]. We conducted CS assays with a Beckman DU 7400 spectrophotometer (Beckman Coulter, Inc., Fullerton, CA) at 39°C. For each assay we collected 2–3 min of data prior to adding substrate (oxaloacetate) to determine control values, followed by 5 min after the addition of substrate. For CS assays, control activities were negligible, so we used only values after addition of oxaloacetate, without subtracting control values, for subsequent analyses. We followed the change in absorbance at 412 nm, using a molar extinction coefficient of 13.6 for activity calculations. We report CS activities as mean mass-specific activity (µmoles · min^−1^·g ^−1^), but we also calculated total CS activities as mass-specific activity * right pectoralis wet mass (g)*2.

### Statistics

We present data as means ± s.e.m., unless otherwise noted. We used least squares linear regression of log_10_-transformed M_b_ vs. log_10_-transformed pectoralis muscle mass, combined flight and leg muscle masses, and heart mass to determine if tissue masses were correlated with M_b_. For these regressions, we used M_b_ − organ mass (both sides combined if muscles are paired) for the M_b_ term to avoid statistical problems associated with part-whole correlations [51]. We also performed least squares regressions of M_b_ vs. pectoralis mass-specific (per gram of muscle) and total (mass-specific activity X muscle mass) CS activities for both raw and log_10_-transformed data to determine if enzyme activities were significantly correlated with M_b_. If we found significant allometric correlations for any of these variables, we calculated residuals from allometric equations for subsequent analyses. We used least squares linear regression to examine correlations of raw values (if allometric equations were not significant) or allometric residuals (if allometric correlations were significant) of M_sum_ vs. tissue masses and mass-specific and total CS activities. Finally, we conducted multiple regressions for both species, with log M_sum_ as the dependent variable and log M_b_, log pectoralis mass, log heart mass and log mass-specific pectoralis CS activity as independent variables. We accepted statistical significance as *P* ≤ 0.05.

## Results

### House Sparrows

**Figure 1:**
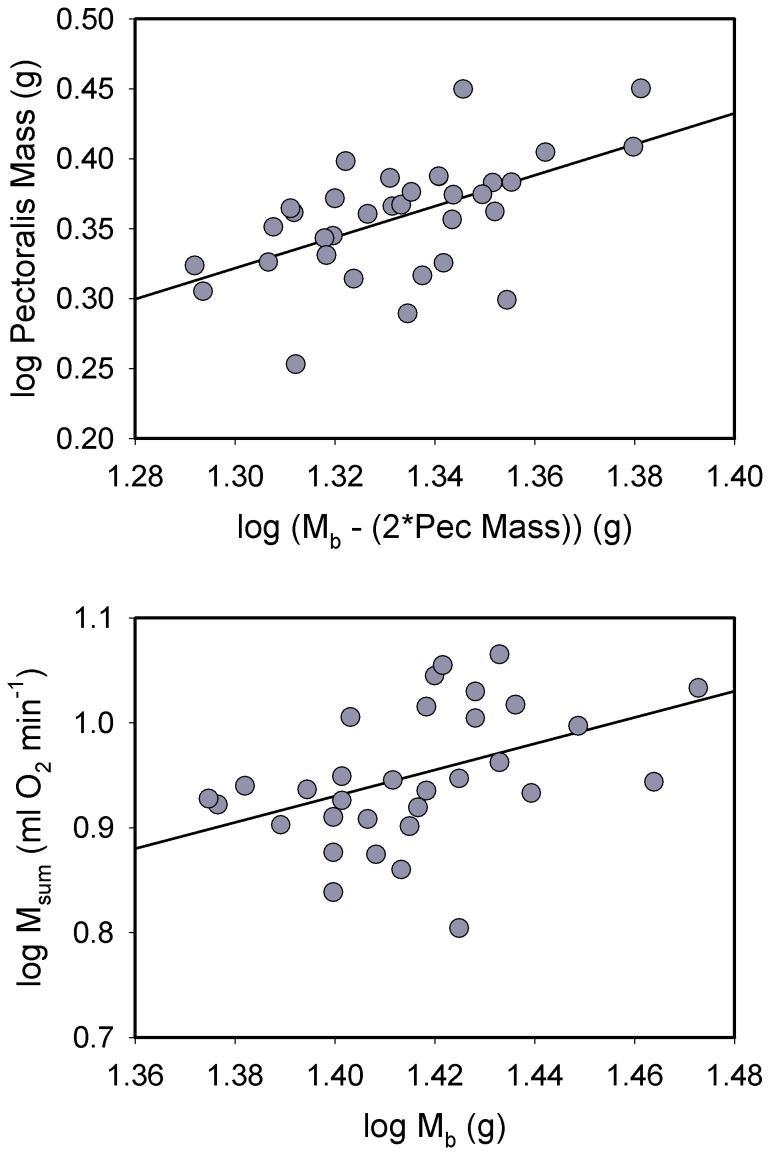
Allometric regressions for log_10_ pectoralis muscle mass (top) and log_10_ M_sum_ (bottom) vs. log_10_ body mass (M_b_) in house sparrows. For the pectoralis mass regression, we used M_b_ – pectoralis muscle mass (for both halves) for the M_b_ term to avoid problems with part-whole correlations [51]. Both regressions were significant and positive (see text for statistics). Regression equations were: log pectoralis mass = −1.118+ (1.108 * log [M_b_ − 2*pec]); log M_sum_ = −0.824+ (1.253 * log M_b_).

**Figure 2:**
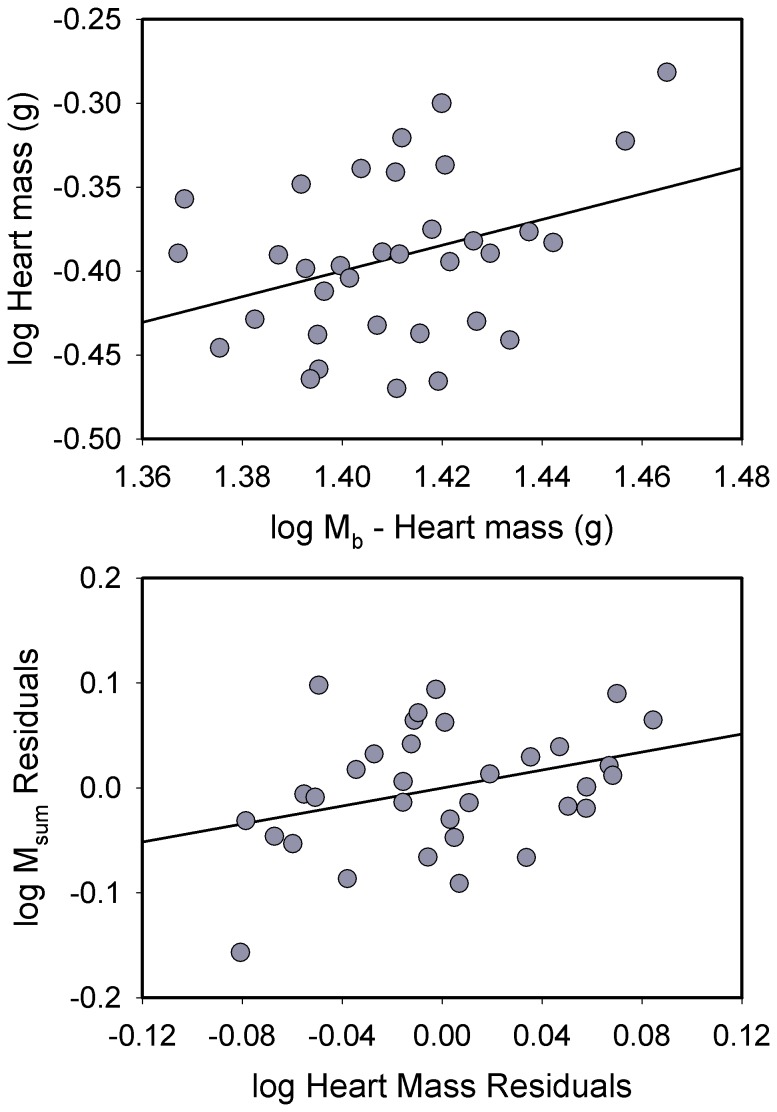
Allometric regression for log_10_ heart mass vs. log_10_ body mass (M_b_) (top) and regression of residuals from allometric regressions for heart mass and M_sum_ (bottom) for house sparrows. The allometric regression was significant and positive and the residual regression was nearly so (*P* = 0.054, see text for additional statistics). Regression equations were: log heart mass = −1.471+ (0.765 log [M_b_ – Heart mass]); log M_sum_ residuals = −0.00007+ (0.428 * log heart mass residuals).

Log_10_ M_sum_ was significantly positively correlated with log_10_ M_b_ (*R^2^* = 0.186, *P* = 0.012) for house sparrows (Fig. 1). Pectoralis mass (Fig. 1; *R^2^* = 0.313, *P*<0.001) and combined flight and leg muscle mass (*R^2^* = 0.357, *P*<0.001) were both significantly positively correlated with M_b_ for log_10_-transformed data. In addition, log_10_ heart mass was significantly positively correlated with log_10_ M_b_ (Fig. 2; *R^2^* = 0.125, *P* = 0.040).

**Figure 3:**
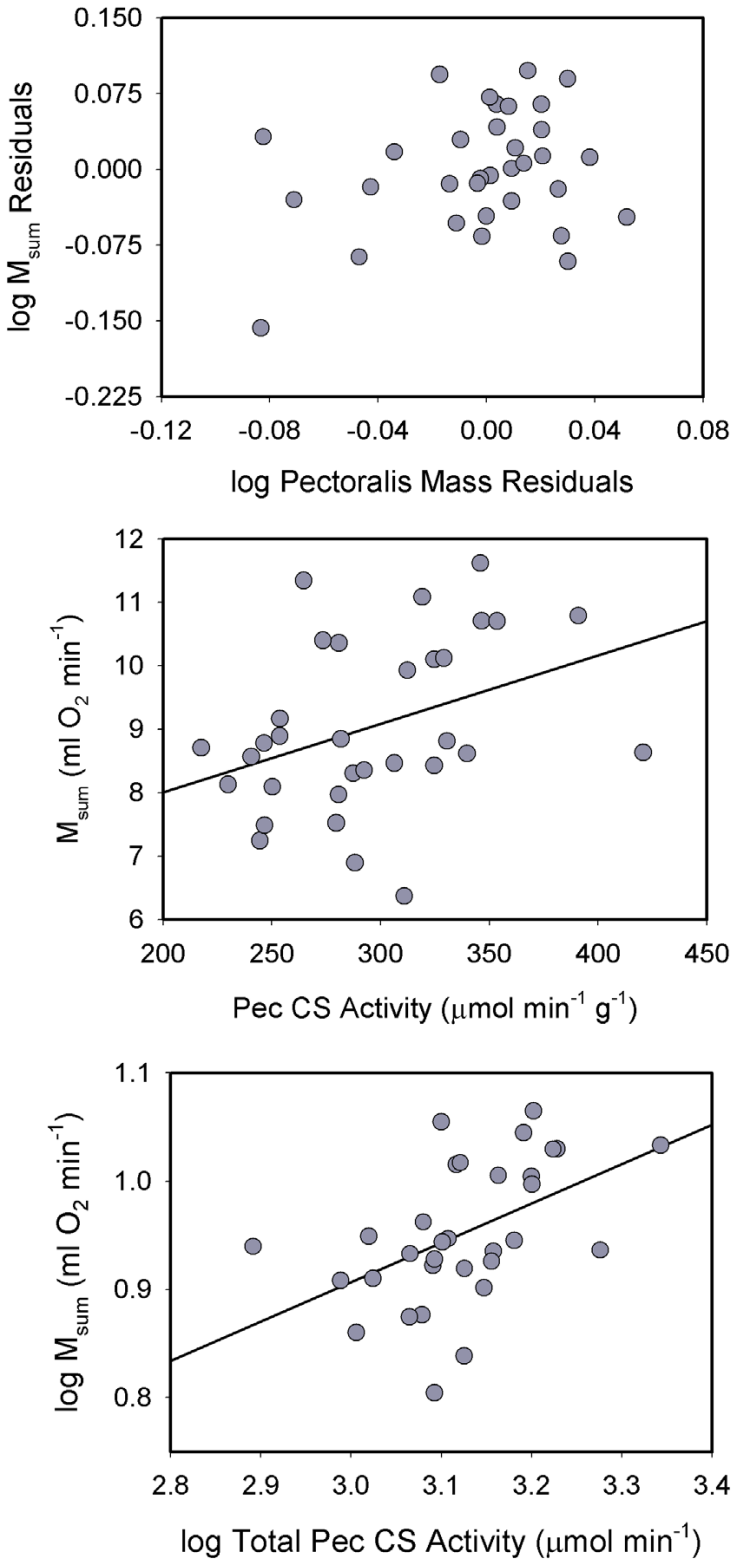
Regressions of residuals of log_10_ pectoralis mass vs. log_10_ M_sum_ (top), mass-specific pectoralis citrate synthase (CS) activity vs. M_sum_ (middle), and log_10_ total pectoralis CS activity vs. log_10_ M_sum_ (bottom) for house sparrows. After adjusting for body mass, pectoralis muscle mass was not significantly correlated with M_sum_. However, both mass-specific and total pectoralis CS activity were significantly positively correlated with M_sum_ (see text for statistics). Regression equations were: M_sum_ = 5.581+ (0.0108 * Pec CS); log M_sum_ = −0.186+ (0.364 * log Total Pec CS).

Raw pectoralis mass was significantly positively correlated with M_sum_ for sparrows (*R^2^* = 0.186, *P* = 0.012). However, allometric residuals of pectoralis mass were not significantly correlated with allometric residuals for M_sum_ (Fig. 3), indicating that the correlation between raw pectoralis mass and M_sum_ was driven by variation in M_b_. Similarly, allometric residuals for combined flight and leg muscle mass were not significantly correlated with M_sum_ residuals. In contrast, allometric residuals for heart mass were nearly significantly positively correlated with M_sum_ residuals (Fig. 2; *R^2^* = 0.115, *P* = 0.054), suggesting that birds with large hearts for their body mass also had higher mass-independent M_sum_.

Neither raw nor log_10_-transformed pectoralis mass-specific or total CS activities were significantly correlated with M_b_, although the regression for total CS activity approached significance (*R^2^* = 0.091, *P* = 0.088). Consequently we used raw pectoralis CS values for correlations with M_sum_. Both mass-specific (*R^2^* = 0.143, *P* = 0.033) and total (*R^2^* = 0.186, *P* = 0.012) pectoralis CS activities were significantly positively correlated with M_sum_ in sparrows (Fig. 3). Because the allometric regression for total pectoralis CS activity approached significance, we also tested for correlations between residuals for total CS and M_sum_. Pectoralis total CS residuals were also significantly positively correlated with M_sum_ residuals (*R^2^* = 0.138, *P* = 0.036) for sparrows.

The multiple regression of all independent variables (log M_b_, log pectoralis mass, log heart mass, log mass-specific pectoralis CS activity) against log M_sum_ was significant (*R^2^* = 0.321, *P* = 0.028) for house sparrows. However, none of the individual variables in the multiple regression were significantly associated with log M_sum_ (all *P*>0.259).

### Dark-eyed Juncos

**Figure 4:**
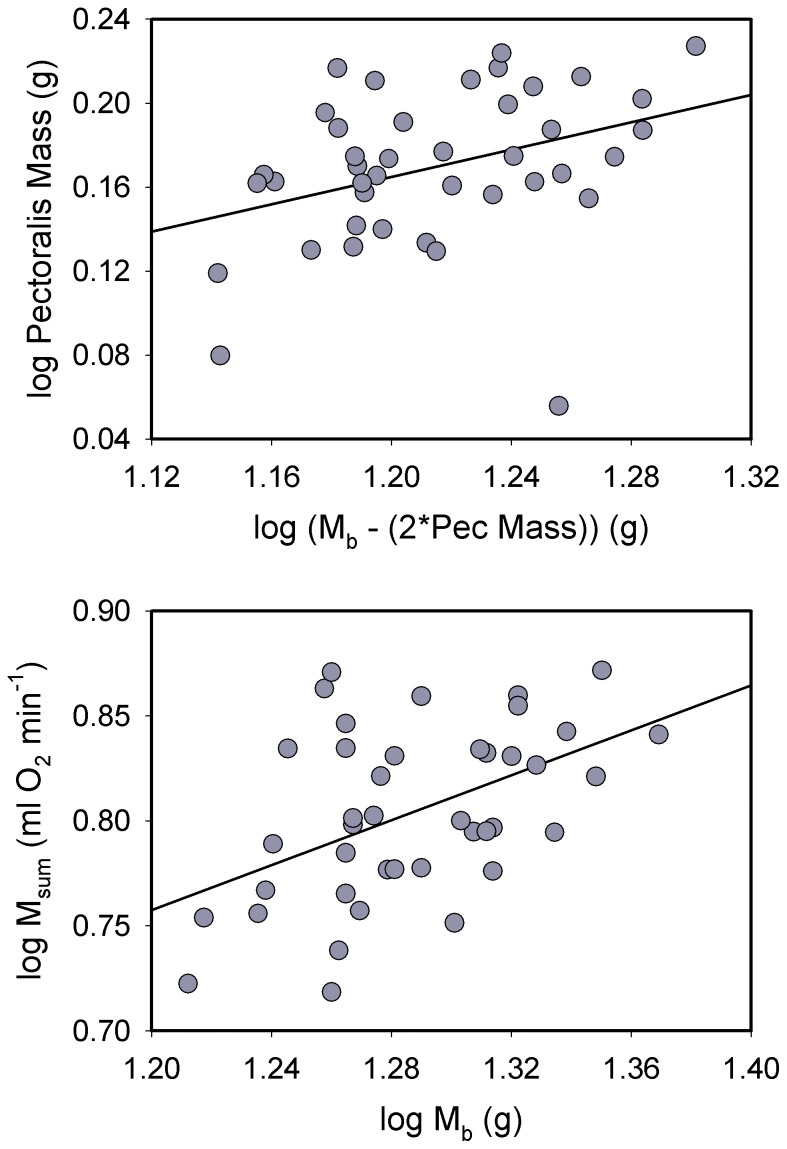
Allometric regressions for log_10_ pectoralis muscle mass (top) and log_10_ M_sum_ (bottom) vs. log_10_ body mass (M_b_) in dark-eyed juncos. For the pectoralis mass regression, we used M_b_ – pectoralis muscle mass (for both halves) for the M_b_ term to avoid problems with part-whole correlations [51]. Both regressions were significant and positive (see text for statistics). Regression equations were: log pectoralis mass = −0.408+ (0.485 * log [M_b_ − 2*pec]); log M_sum_ = 0.115+ (0.536 * log M_b_).

**Figure 5:**
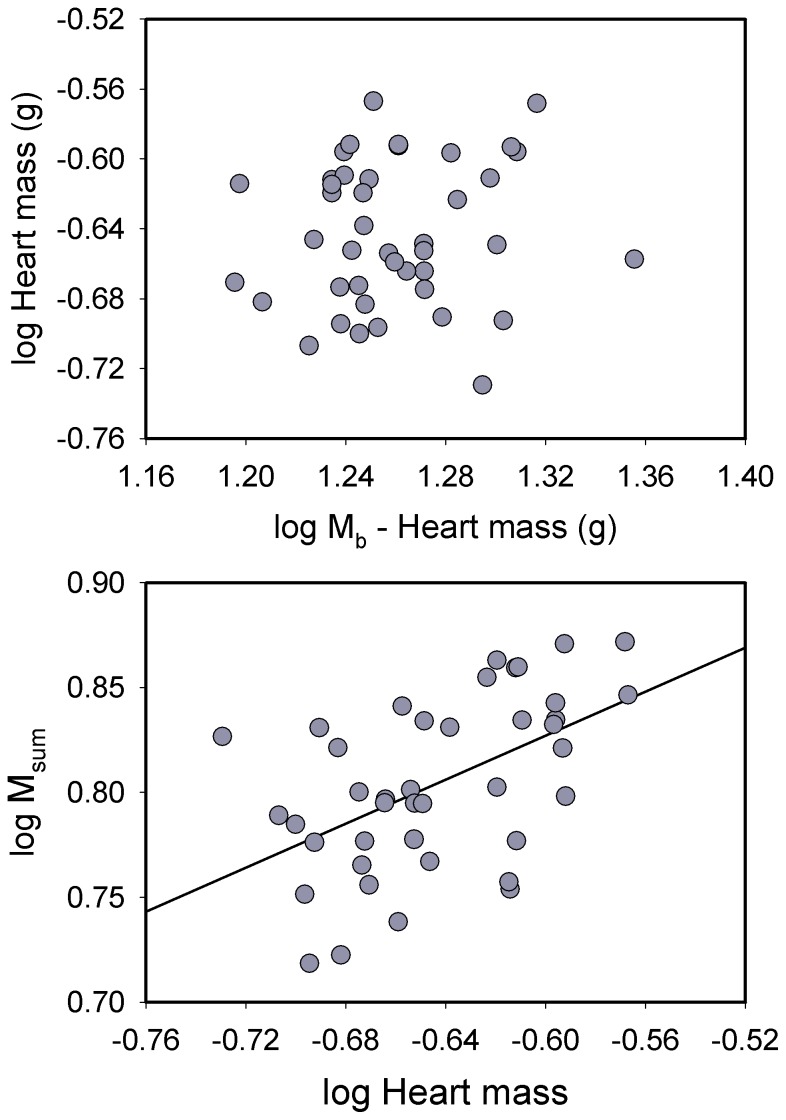
Allometric regression for log_10_ heart mass vs. log_10_ body mass (M_b_) (top) and regression of log_10_ heart mass and log_10_ M_sum_ (bottom) for dark-eyed juncos. Heart mass was not significantly correlated with body mass (see text for additional statistics), but log heart mass was significantly positively correlated with log M_sum_ according to the equation log M_sum_ = 1.142+ (0.524 * log heart mass).

Log_10_-transformed body mass in juncos was significantly positively associated with log_10_ pectoralis muscle mass (Fig. 4; *R^2^* = 0.205, *P* = 0.003) and log_10_ combined flight and leg muscle mass (*R^2^* = 0.146, *P* = 0.013). However, log_10_ M_b_ was not significantly correlated with log_10_ heart mass (Fig. 5). Log_10_-transformed M_b_ was significantly positively associated with log_10_ M_sum_ for juncos (Fig. 4; *R^2^* = 0.234, *P* = 0.001).

**Figure 6:**
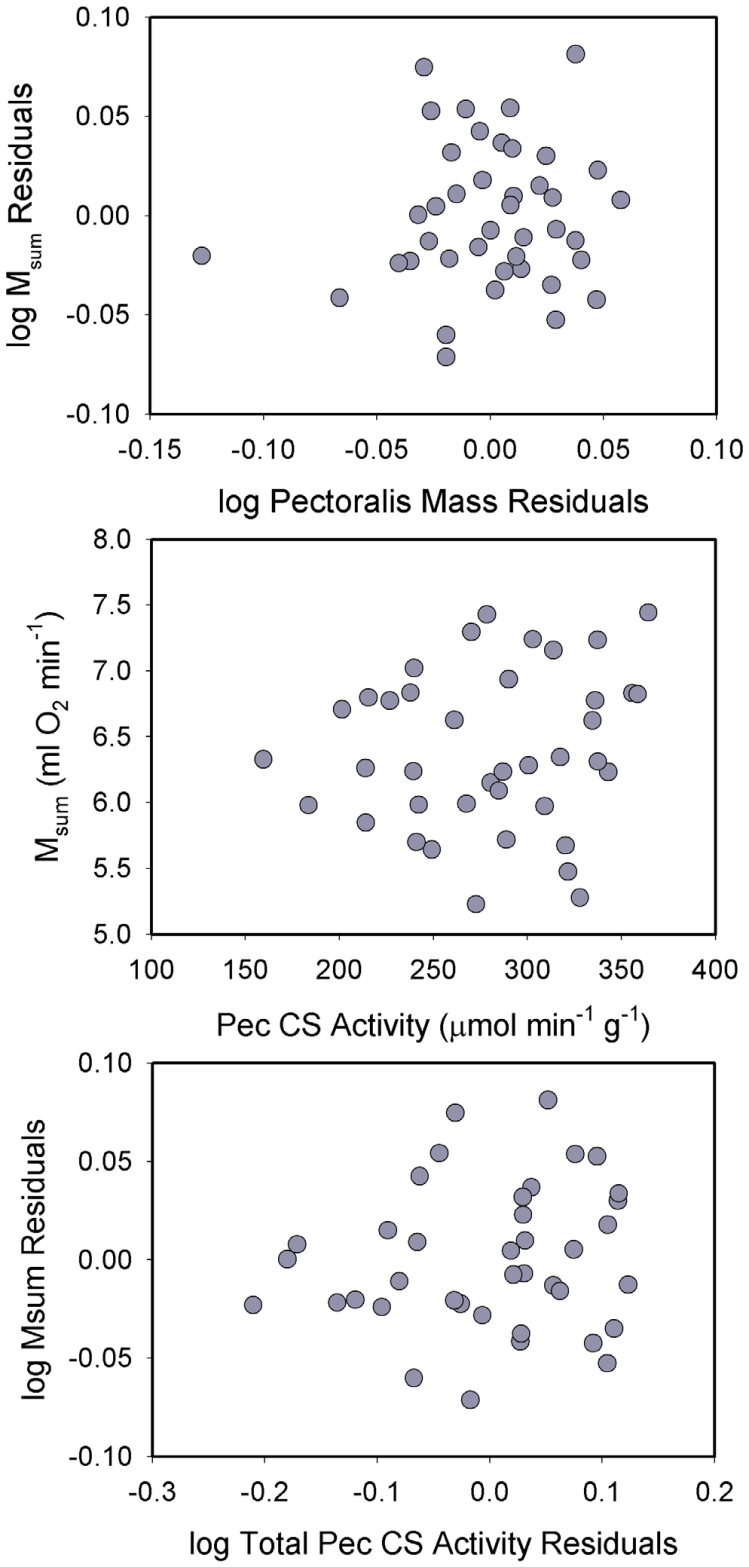
Regressions of residuals of log_10_ pectoralis mass vs. log_10_ M_sum_ (top), mass-specific pectoralis citrate synthase (CS) activity vs. M_sum_ (middle), and log_10_ total pectoralis CS activity vs. log_10_ M_sum_ (bottom) for dark-eyed juncos. None of the correlations were statistically significant.

Raw pectoralis mass was weakly positively correlated with raw M_sum_, with the correlation almost reaching significance (*R^2^* = 0.093, *P* = 0.053). However, allometric residuals for pectoralis mass and M_sum_ were not significantly correlated (Fig. 6), indicating that the correlation between raw pectoralis mass and M_sum_ was driven by variation in M_b_. Likewise, allometric residuals for combined flight and leg muscle masses and M_sum_ were not significantly correlated for juncos. Heart mass, however, was significantly positively correlated with M_sum_ in juncos, both for raw (*R^2^* = 0.291, *P*<0.001) and log_10_-transformed values (Fig. 5; *R^2^* = 0.272, *P*<0.001).

Mass-specific pectoralis CS activity was not significantly correlated with M_b_ in juncos, for either raw or log_10_-transformed values. Total CS activity in pectoralis, however, was positively correlated with M_b_, significantly so for raw values (*R^2^* = 0.116, *P* = 0.034) and nearly significantly so for log-_10_ transformed data (*R^2^* = 0.083, *P* = 0.075). Neither raw (Fig. 6) nor log_10_-transformed mass-specific CS activities were significantly correlated with M_sum_. Likewise, allometric residuals for total pectoralis CS activity were not significantly correlated with M_sum_ residuals (Fig. 6).

The multiple regression of all independent variables (log M_b_, log pectoralis mass, log heart mass, log mass-specific pectoralis CS activity) against log M_sum_ was significant (*R^2^* = 0.469, *P*<0.001) for juncos. Among independent variables in the multiple regression, both log heart mass (*P*<0.001) and log M_b_ (*P* = 0.013) were significant effectors of log M_sum_.

## Discussion

Both house sparrows and dark-eyed juncos in this study showed a positive correlation of pectoralis mass with M_sum_, but this correlation disappeared after accounting for the effects of body mass, as allometric residuals of pectoralis mass and M_sum_ were not significantly correlated for either species. This indicates that the relationship between pectoralis mass and M_sum_ is driven by variation in body mass, with large birds possessing large pectoralis muscles and high M_sum_, so pectoralis mass is likely important to total (per bird) M_sum_. However, the absence of significant correlations between residual pectoralis mass and residual M_sum_ for both species in the current study contrasts with results from several other avian studies, where pectoralis muscle mass was positively correlated with maximum metabolic output independent of body mass variation, such that birds with larger pectoralis muscles for their body size also had relatively higher maximum metabolic output [23], [25], [34], [36], [37]. Moreover, seasonal or migration-induced changes in pectoralis muscle mass or size are usually accompanied by similar changes in maximum metabolic output [5], [30]. However, increments of M_sum_ associated with winter-acclimatization or cold-acclimation in small birds can occur without corresponding increases in pectoralis muscle mass, although such changes are not necessarily consistent among studies, even for the same species. For example, American goldfinches (*Spinus tristis*) from Michigan showed higher organismal M_sum_ in winter than in summer [14], despite an absence of seasonal variation in pectoralis muscle mass [32]. In contrast, American goldfinches from South Dakota showed both higher organismal M_sum_ [18] and larger pectoralis muscle masses [33] in winter. Black-capped chickadees (*Poecile atricapillus*) exhibit higher M_sum_ in winter than in summer [16], [52], but summer to winter variation in pectoralis muscle mass apparently varies among years [29], [33]. Finally, dark-eyed juncos show winter increments of M_sum_ and pectoralis muscle mass [15], [27], whereas cold-acclimation in juncos promoted higher M_sum_ without corresponding changes in pectoralis muscle mass [42]. Thus, flexibility of M_sum_ in small birds is often, but not universally, associated with correlated variation in pectoralis muscle mass, suggesting that other mechanisms are available for birds to produce elevated M_sum_.

In this study, birds were collected and measured only during a single season, winter for juncos and late spring for sparrows. Thus, the study design measured only mechanistic correlates of variation in M_sum_ among individual birds within a season, rather than across the entire annual cycle. Variation in M_sum_ is greater over the entire annual cycle than during a single season, so the possibility exists that variation in M_sum_ among individual birds in the current study was not sufficient to detect correlations with pectoralis mass, and that pectoralis mass could function as a driver of M_sum_ variation across seasons, but not within seasons for these species. However, several bird species show significant or nearly significant within-season correlations of residual pectoralis size and residual M_sum_ [24], [25], [34]. Moreover, juncos on the different temperature/photoperiod treatments showed variation in mean M_sum_ of up to 19% among treatment groups, a value only slightly lower than natural seasonal variation in M_sum_ in this species [42]. Thus, the absence of significant correlations of pectoralis mass residuals with M_sum_ residuals suggests that variation in pectoralis muscle mass is less important to M_sum_ variation in the current study species than in several other bird species.

Because other skeletal muscles may also contribute to shivering thermogenesis in small birds [53], variation in the masses of muscles such as supracoracoideus or leg might contribute to variation in M_sum_. Indeed, some birds do show winter increases in masses of supracoracoideus muscles, but winter increments in supracoracoideus and leg muscle masses are not common nor consistent components of the winter phenotype in small birds [29], [33], [53]. In addition, leg muscle masses were not correlated with maximum metabolic output in house sparrows [36] or red junglefowl [37]. Given that combined flight and leg muscle masses were not significantly correlated with M_sum_ for either species in the current study, our results are consistent with these former studies and suggest that sizes of skeletal muscles other than the pectoralis are not prominent drivers of maximum metabolic output in volant birds generally.

In addition to muscle mass variation, another potential driver of variation in M_sum_ in birds is the cellular metabolic intensity of muscles, particularly the pectoralis [5], [26]. Cellular metabolic intensity is usually estimated by measuring activities of citrate synthase or cytochrome c oxidase, key regulatory enzymes in aerobic metabolic pathways. Activities of these enzymes may vary with changing energy demand in small birds, such as winter acclimatization or migration, but such changes are not universally associated with these periods of the annual cycle [5], [26]. For example, pectoralis CS or COX activities increase with winter acclimatization in black-capped chickadees [38], [54] and Chinese bulbuls (*Pycnonotus sinensis*) [55], but remain seasonal stable in house sparrows and white-breasted nuthatches (*Sitta carolinensis*) [38], northern cardinals (*Cardinalis cardinalis*) [30], American goldfinches [54], and rufous-collared sparrows (*Zonotrichia capensis*) [39]. Similarly, recent studies reveal that cellular metabolic intensity increases with migratory condition in white-throated sparrows (*Zonotrichia albicollis*) [56], but other species such as red knots (*Calidris canutus*) [57] and warbling vireos (*Vireo gilvus*), and yellow (*Setophaga petechia*) and yellow-rumped (*Setophaga coronata*) warblers [54] fail to show similar migration-induced variation. Indeed, opposing trends may occur within the same species, as Driedzic et al. [58] documented no variation in pectoralis CS or COX activities with migration in semipalmated sandpipers (*Calidris pusilla*), but Maillet and Weber [59] found that CS activity increased with fattening in preparation for migratory flights in this same species. Other patterns of pectoralis CS variation may also occur. For example, Box et al. [60] documented elevated pectoralis CS activity during spring relative to other seasons in the non-migratory superb fairy-wren (*Malurus cyaneus*) from mild-temperate climates in Australia, likely associated with increased energy expenditure associated with breeding. However, this seasonal variation in pectoralis CS activity in fairy-wrens was not associated with similar seasonal variation in metabolic rates [61]. Thus, the generalization for small birds seems to be that variation in cellular metabolic intensity in pectoralis occurs in response to increasing energy demands in some species or individuals but not in others, so pectoralis cellular metabolic intensity appears to be an inconsistent driver of variation in maximal metabolic output with changing energy demands [5].

Cellular metabolic intensity may also correlate positively with variation in maximal metabolic output in birds, but again, such variation with maximum metabolic output is not universal among birds. For example, pectoralis CS activity was not correlated with M_sum_ in American goldfinches [34]. In addition, variation in mass-specific CS activity was unrelated to differences in maximum exercise metabolic rates between juvenile and adult house sparrows [40]. Gender differences in the relationship between pectoralis CS activity and maximum exercise metabolic rates occurred for red junglefowl, with males demonstrating a positive relationship, but females showing no relationship [37]. In our study, house sparrows showed significant positive correlations of pectoralis CS activity with M_sum_, but juncos did not, which underscores the general situation for small birds, where cellular metabolic intensity is an inconsistent driver of variation in maximal metabolic output. In addition, the greater statistical significance for correlations of total than of mass-specific pectoralis CS activity in sparrows in the current study suggests that pectoralis muscle mass also plays a minor role as a driver of M_sum_ in sparrows, despite the absence of significant correlations between residuals of pectoralis mass and M_sum_ in this study.

In contrast to the absence of significant correlations of pectoralis and combined flight and leg muscle masses with M_sum_ in both species in this study, and the inconsistent correlations of cellular metabolic intensity with M_sum_, both species showed positive correlations of allometric residuals of heart mass with allometric residuals of M_sum_, which were marginally significant for sparrows in simple linear regression analyses and highly significant for juncos for both simple and multiple regression analyses. This result suggests that variation in heart mass is an important driver of M_sum_ variation in small birds generally. The highly significant correlation in juncos is consistent with the finding that heart mass varied significantly with cold-acclimation, along with M_sum_, in these same individuals [42]. In addition, heart mass is a consistent contributor to the winter phenotype in small birds, with winter heart mass higher than summer heart mass in Eurasian tree sparrows (*Passer montanus*) [62], [63], house sparrows and white-breasted nuthatches [29], American goldfinches [33] and Chinese bulbuls [56]. Black-capped chickadees also regularly show winter increments of heart mass relative to summer [29], [35], but this winter increment apparently does not occur in all winters for chickadees [33]. Increases in heart mass are also a common and consistent element of the migratory phenotype in birds [64]–[68], suggesting that increases in heart mass are associated with periods of increasing energy demand throughout the annual cycle.

Heart mass might be expected to be correlated with both basal (BMR) or resting (RMR) metabolic rates and maximum metabolic output, because of its activity both during rest and during activities requiring elevated energy demands. Indeed, heart mass is often positively correlated with metabolic rates in birds, but is apparently more regularly correlated with maximal than minimal metabolic output. For example, although heart mass is positively correlated with BMR or RMR in some species [55], a number of other species fail to show such correlations [36], [37], [69], [70]. Maximum metabolic output during exercise [36], [37] or shivering [35], however, is consistently positively related to heart mass. This suggests that modulation of heart mass is an important contributor to variable metabolic phenotypes in birds generally. The mechanisms responsible for regulating such variation in heart mass are currently unknown in birds, but gene expression of myostatin and its metalloproteinase activators in heart muscle is upregulated along with heart mass in winter in some small bird species [42], suggesting that myostatin is a potential regulator. Verification of this mechanism for promoting heart mass changes in birds generally and the potential involvement of other regulatory pathways will require further research.

In conclusion, our data are consistent with previous data in suggesting that different species modify different physiological parameters to accomplish metabolic flexibility. Previous studies suggest that changes in pectoralis muscle size are common drivers of flexibility in maximum metabolic output in birds [34], [36], [37], and bigger birds had larger pectoralis muscles and higher M_sum_ for both juncos and sparrows, but these differences were associated with variation in M_b_, not with variation in pectoralis muscle mass independent of M_b_. Pectoralis muscle mass adjusted for body size was not significantly associated with M_sum_ for either juncos or house sparrows in the current study, so modulating pectoral muscle size independent of M_b_ changes is evidently not a universal driver of metabolic flexibility in birds. Similarly, variation in pectoralis muscle myocyte metabolic intensity contributes to variation in maximal metabolic output in some birds, but not in others [5], [26], and our study highlights this point as we found a positive correlation of cellular metabolic intensity with M_sum_ for sparrows, but not for juncos. Variation in heart or cardiovascular organ masses is consistently positively correlated with maximal, but not minimal, metabolic outputs in birds [5], [35], including both sparrows and juncos in the current study, suggesting that cardiovascular variation may be among the most consistent drivers of flexibility in maximum metabolic output in birds. However, heart mass is not as strong a predictor of maximal metabolic output as pectoralis mass in some species [36], [37]. Thus, these data underscore the diversity of mechanistic responses contributing to metabolic flexibility in birds and suggest that these mechanistic responses may vary among species and, potentially, within species experiencing environmental or ecological conditions which promote flexible metabolic responses.

## Supporting Information

Data File S1 Raw data file for house sparrows (*Passer domesticus*) in this study. Abbreviated column titles: body mass (Mb); pectoralis mass (Pec); supracoracoideus mass (Scc); leg muscle mass (Leg); heart mass (Hrt); summit metabolic rate (Msum); pectoralis CS activity (Pec CS); total CS activity (Tot CS = pectoralis mass X mass-specific CS activity); combined masses of paired pectoralis, supracoracoideus and leg muscles (All MM).
(XLSX)

Data File S2 Raw data file for dark-eyed juncos (*Junco hyemalis*) in this study. Abbreviated column titles are the same as for Data File S1.
(XLSX)
